# Home Parenteral Support in Chronic Intestinal Failure—First Results from a Pioneer Portuguese Intestinal Failure Center

**DOI:** 10.3390/nu16223880

**Published:** 2024-11-14

**Authors:** Ivo Mendes, Francisco Vara-Luiz, Carolina Palma, Gonçalo Nunes, Maria João Lima, Cátia Oliveira, Marta Brito, Ana Paula Santos, Carla Adriana Santos, Jorge Fonseca

**Affiliations:** 1 GENE—Artificial Feeding Team, Gastroenterology Department, Hospital Garcia de Orta, 2805-267 Almada, Portugal; franciscovaraluiz@gmail.com (F.V.-L.); carolinamrpalma@gmail.com (C.P.); goncalo.n@hotmail.com (G.N.); jorgedafonseca@gmail.com (J.F.); 2 Egas Moniz Center for Interdisciplinary Research (CiiEM), Egas Moniz School of Health & Science, 2829-511 Almada, Portugal

**Keywords:** intestinal failure, home parenteral support, home parenteral nutrition, home parenteral hydration, short bowel syndrome

## Abstract

Background/Objectives: Home parenteral support (HPS) is the core of chronic intestinal failure (IF) treatment. For legal reasons, HPS in Portugal lags behind other European countries, and only a few patients were taken care of at home by nurses. Now, the legislation has changed, allowing patient self-care. The authors report their pioneer experience as the largest Portuguese IF center, evaluating the underlying conditions leading to IF, HPS nutritional impact, HPS-related complications and survival. Methods: This is a retrospective study including IF patients who underwent HPS in a Portuguese IF center. The data included demographics, underlying conditions, IF types, HPS duration, BMI at the beginning and end of HPS/follow-up, complications, microbiological agents of infectious complications and current status (deceased or alive with/without HPS). Survival was calculated until death or September 2024. Results: A total of 23 patients (52.2% female, mean age 57.3 years), all with type III IF, were included. Short bowel syndrome (SBS) was the most common cause of IF (69.6%). Of the included patients, 78.3% received home parenteral nutrition; the others received home parenteral hydration. The mean BMI increased significantly, from 19.1 kg/m^2^ to 22.5 kg/m^2^ (*p* < 0.001). Two patients received Teduglutide. The most common complication was catheter-related bloodstream infection (2.5/1000 catheter days). The complications did not increase with patient self-care. At the end of follow-up, 21.7% of patients remained on HPS, 34.8% were alive without HPS, and 43.5% died. The average survival was 43.4 months. One death (4.35%) was attributable to HPS-related complications. Conclusions: The conditions underlying IF varied, with SBS being the most frequent condition. HPS improved the BMI, allowing considerable survival. Despite the complications and one attributable death, HPS was safe, even when relying on patient self-care.

## 1. Introduction

Intestinal failure (IF) is defined as a reduction in gut function below the minimum necessary for absorption of macronutrients and/or water and electrolytes, such that intravenous supplementation (IVS) is required to maintain health and/or growth. A reduction in absorption that does not require IVS can be defined as intestinal insufficiency/deficiency [1,2,3], although this concept is not completely accepted by all authors.

Several criteria can be used to classify IF, reflecting the complexity of this condition [1,4,5] (Table 1). Functionally, IF can be divided into three types: type I—an acute and short-term form of IF most often seen post-operatively, requiring temporary IVS and resolving spontaneously; type II—a prolonged subacute IF most often seen in metabolically unstable patients with a high-output stoma or enteric fistulas; type III—a chronic condition in metabolically stable patients requiring IVS over months/years, being reversible or irreversible. Occasionally, type II IF can progress to type III [1]. From a clinical perspective, the severity of IF can be classified into eight categories according to the type and volume of the IVS required by patients. From a pathophysiological perspective, there are five major mechanisms leading to IF: short bowel, intestinal fistula, extensive small bowel mucosal disease, intestinal dysmotility and mechanical obstruction. Frequently, IF patients have more than one underlying pathophysiological mechanism. Furthermore, short bowel syndrome (SBS), the most frequent cause of IF in adult patients, can be divided into three major morphological/anatomical groups: end jejunostomy (type 1), jejunocolic anastomosis (type 2) and jejunoileocolic anastomosis (type 3). This classification must not be confused with the three types of IF described above.

IF is a complex and debilitating condition best managed by a multidisciplinary team with differentiated expertise [6]. As long-term parenteral support is indicated for patients with prolonged/chronic intestinal failure, home parenteral support (HPS)—home parenteral nutrition (HPN) and/or home parenteral hydration (HPH)—remains the core treatment for these patients (mostly type III IF but also some patients with type II IF) [7].

A broad range of HPS-related complications can affect IF patients, frequently leading to hospitalizations, reduced quality of life, higher health-related costs and even mortality. Metabolic complications are multifactorial and comprise fluid, electrolyte and acid-base balance abnormalities, metabolic bone disease, intestinal-failure-associated liver disease (IFALD), renal function impairment and stones, gallbladder sludge and stones, among others [8]. As HPS is dependent on a well-functioning central venous access, catheter-related complications can arise both from its insertion and use. They comprise complications like catheter-related infections, thrombosis and dysfunction [9].

Although type I IF is frequent, types II and III are rare [4]. Twenty-five years ago, the prevalence of chronic IF was typically reported as fewer than 10 in 1,000,000 people in European countries [10], but more recent studies support rising prevalences [11,12,13,14,15]. However, the real prevalence is not well known, displaying a very wide international variation. Such variety can reflect the use of different methodologies and definitions. In Portugal, the data on IF are scarce, and there is a lack of a national registry, as well as no formal network organization between health institutions to concentrate the management of these patients in IF-experienced centers [16].

The implementation of HPS in Portugal encountered legal barriers for a long period of time, causing it to lag significantly behind other European countries. Our team gained earlier experience with HPS compared to other Portuguese centers because our hospital pioneered the establishment of domiciliary hospitalization units in 2015. However, only patients living within the domiciliary hospitalization unit’s designated area could undergo HPS, which was administered by the hospital nurses. In 2020, the Portuguese health authorities (Direção Geral da Saúde) developed a new directive (“Norma 017/2020”) that regulated the conditions for home IF management [17]. Subsequently, HPS became more independent from domiciliary hospitalization, and we witnessed a paradigm change: on the one hand, we began receiving patients referred from other regions; on the other hand, we started to train patients toward HPS self-administration before discharge or to hospitalize patients from other areas to undergo comprehensive training carried out by our multidisciplinary team. With these new strategies, we could ensure that patients learn and master every aspect of therapy administration, enabling full independence and minimizing the risk of complications. Overall, this new legal background is leading to an increasing number of IF patients being now treated in an ambulatory setting in our center and around the country [16].

We outline our experience with HPS in patients with IF, amounting to a decade of experience as the largest intestinal failure center in Portugal. The main goals of this study were to review the different underlying conditions that lead to IF; to analyze the impact of HPS on the nutritional status; to determine the survival for IF patients; to assess for HPS-related complications and compare the complications before and after the paradigm change had occurred.

## 2. Materials and Methods

The authors conducted a retrospective analysis of all patients with IF who had undergone HPS (HPN and/or HPH) or were under HPS at the time of data collection (September 2024) followed in a single large tertiary Portuguese hospital. All IF patients were managed by the institutional Artificial Feeding Team (GENE), a multidisciplinary nutrition support team composed of medical doctors (including gastroenterologists and digestive surgeons), pharmacists, dietitians and nurses.

The following data were collected for each patient: age; gender; underlying condition that led to IF; number of days per week under IVS; energy from HPN and/or volume of HPH for the majority of the HPS period (after the initial stabilization and before starting HPS withdrawal); body mass index (BMI) at the beginning of HPS and at the end of HPS (or at the end of follow-up); HPS-related complications requiring hospitalization; the isolated microbiological agent responsible for infectious complications; patient status at the time of data collection (deceased, alive with HPS or alive without HPS). Patients under 65 years of age were categorized according to BMI as underweight (BMI < 18.5 kg/m^2^), normal (BMI 18.5–24.9 kg/m^2^), overweight (BMI 25–29.9 kg/m^2^) or obese (BMI ≥ 30 kg/m^2^). Patients 65 years of age or more were categorized according to BMI as underweight (BMI < 22 kg/m^2^), normal (BMI 22–26.9 kg/m^2^), overweight (BMI 27–29.9 kg/m^2^) or obese (BMI ≥ 30 kg/m^2^). Patients were also classified according to the functional, clinical and pathophysiological classifications of IF, as well as morphological/anatomical classification for SBS patients. Complications before and after the paradigm change (with the development of “Norma 017/2020”) were categorized. For the deceased patients, the date and cause of death were also collected. Survival was calculated in months from the beginning of HPS until the date of death or until the end of follow-up (September 2024).

Statistical analysis was performed using the Statistical Package for Social Sciences (IBM SPSS^®^ Statistics, version 29.0). Categorical variables are presented as absolute and relative frequencies, and continuous variables are presented as means (standard deviations) and medians (interquartile ranges). To compare BMI at the beginning and at the end of follow-up/end of HPS, the Wilcoxon test was used after testing for normality using the Kolmogorov–Smirnov test. Inferential statistics were performed at a 5% level of statistical significance.

## 3. Results

A total of 23 patients under HPS were included: 12 females (52.2%) and 11 males (47.8%). The age at the start of HPS ranged from 25 to 90 years (mean 57.3 [16.9], median 59.0 [45.0–67.0]), with 15 patients (65.2%) under 65 years and 8 patients (34.8%) aged 65 years or older. Most patients underwent HPN (*n* = 18, 78.3%), with the remaining patients (*n* = 5, 21.7%) managed only with HPH. All patients had type III IF. Although six (26.1%) patients initially presented with type II IF, requiring multiple hospitalizations and reinterventions, they achieved a sufficient metabolic stability and had a prolonged course that allowed for home management. Regarding severity classification, patients under HPH could be classified as FE1 (*n* = 3) or FE2 (*n* = 2), while HPN patients were classified as PN1 (*n* = 1), PN2 (*n* = 11) or PN3 (*n* = 6). Regarding pathophysiological classification, SBS was the most frequent condition (*n* = 16, 69.6%), mainly type 1 SBS. The conditions underlying surgical interventions that led to SBS were varied: intestinal ischemia (*n* = 4), bowel adhesions leading to intestinal obstruction (*n* = 3), colorectal cancer with surgical complications (*n* = 3), familial adenomatous polyposis (*n* = 2), Crohn’s disease (*n* = 2), incarcerated umbilical hernia (*n* = 1) and intestinal tuberculosis leading to intestinal occlusion (*n* = 1). In most of these patients, multiple surgical procedures were performed, ultimately leading to SBS. Intestinal fistula was the second most common group, with four patients (17.4%) developing enteric fistulas in the post-operative period or Crohn’s disease fistulas. Two patients (8.7%) could be categorized as having intestinal dysmotility due to chronic intestinal pseudo-obstruction (CIPO) and one (4.3%) as having an extensive small bowel mucosal disease due to hypogammaglobulinemic sprue from rituximab therapy. No patients could be categorized as having a mechanical obstruction. Table 2 presents the patient characteristics and distribution according to functional, severity and pathophysiological classifications.

HPS was performed for a minimum of 4 months and a maximum of 139 months, with a mean of 27.6 (31.8) and a median of 14 (9–31). Regarding patient status at the time of data collection, 5 patients (21.7%) were alive under HPS, 8 (34.8%) were alive without HPS, and 10 (43.5%) patients were deceased. Two patients underwent treatment with Teduglutide, a glucagon-like peptide 2 (GLP-2) analog. One of the patients was already in the weaning process, reaching a stable phase receiving HPN only once per week. After six months of treatment with Teduglutide, this patient was able to completely wean off HPN, maintaining only HPH and intravenous electrolytes. Unfortunately, this patient died on the seventh month after Teduglutide therapy was started with an HPS-related complication (further specified). The other patient started this therapy at the moment of data collection—a patient with a past medical history of gynecological cancer with no evidence of recurrence after 5 years.

**Figure 1:**
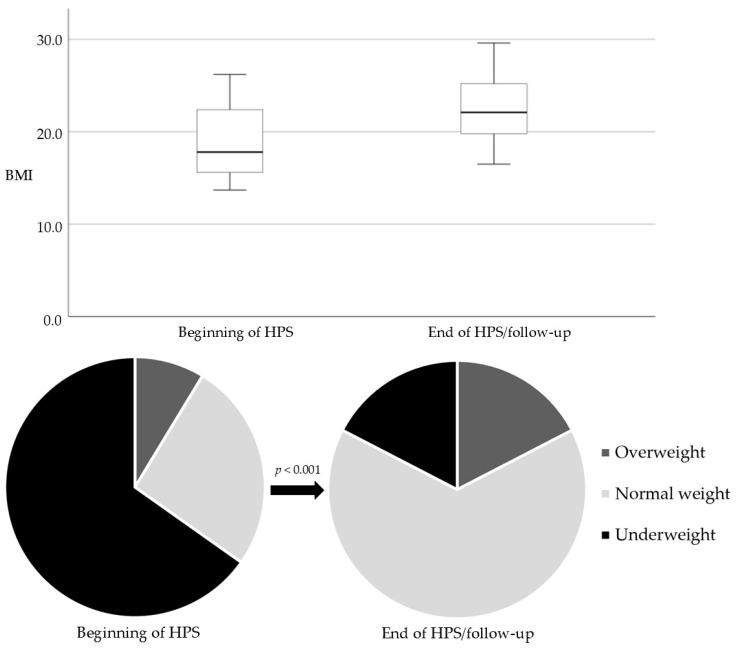
BMI at the beginning of HPS and at the end of HPS/follow-up (boxplots and pie charts).

Before the beginning of HPS, 15 patients were underweight, 6 had normal weight, and 2 were overweight (mean of 19.1 [4.2], median of 17.8 [15.6–22.8]). At the end of HPS/follow-up, 4 patients were underweight, 15 had normal weight, and 4 were overweight (mean of 22.5 [3.6], median of 22.1 [19.6–25.5]). There were no obese patients. The overall BMI improved with HPS (*p* < 0.001) (Figure 1).

Prior to the implementation of “Norma 017/2020”, nine patients (39.1%) received HPS administered by hospital nurses in strict collaboration with domiciliary hospitalization. Afterward, ten patients (43.5%) underwent a self-administration regimen after completing an in-hospital training period. Four patients (17.4%) shifted from one regimen to the other.

Overall, HPS-related complications had an incidence of 4.3/1000 catheter days. Prior to the implementation of “Norma 017/2020” and the subsequent paradigm shift in our hospital, the complication rate was 4.5 per 1000 catheter days, and afterward, it was 4.1 per 1000 catheter days. Catheter-related bloodstream infections (CRSBIs) were the most frequent HPS-related complications, with an incidence of 2.5/1000 catheter days. Gram-positive bacteria accounted for the majority of CRSBIs (59.2%), followed by Gram-negative bacteria (18.4%) and fungi (16.3%), with *Staphylococcus aureus* being the most frequent microbiological agent isolated. In three cases (6.1%), there was no microbiological agent identified. In two cases, the CRSBI was complicated by acute endocarditis. Table 3 summarizes the isolated microbiological agents. Metabolic complications requiring hospitalization had an incidence of 1.2/1000 catheter days. These included complications like the refeeding syndrome and other electrolyte and acid-base balance abnormalities and acute renal failure. Metabolic complications like IFALD and metabolic bone disease were not identified, as most patients were under HPS for a not very long period of time. Other less frequent complications were dysfunction/exteriorization of the catheter (incidence of 0.4/1000 catheter days) and thrombotic complications (incidence of 0.3/1000 catheter days). Table 4 summarizes the incidence of complications. HPS-related complications led to hospitalization in 20 patients (87.0%), with an overall mean of 3.1 hospitalizations per patient and a mean duration of 22.9 days.

There was one death directly attributable to HPS-related complications—a patient with prosthetic aortic and mitral valves who died because of a CRBSI with an abscess-complicated acute endocarditis. Other deaths occurred due to acute infections not related to HPS in six patients and due to comorbidities in three patients.

The minimum and maximum survival in months from the beginning of HPS was 4 and 146, respectively (mean survival of 43.4 (39.9) and median survival of 31 (14–72)).

## 4. Discussion

The present study reflects our experience with HPS in IF patients, focusing on the underlying conditions, nutritional outcomes, complications and survival.

The study included 23 adult patients with chronic IF, representing the largest series of IF patients undergoing HPS followed in a single Portuguese center.

The most common pathophysiological cause of IF in our study was SBS, representing 69.6% of the cases. This finding is similar to those reported in the literature, confirming SBS as the leading cause of chronic IF [1,4]. The underlying conditions that ultimately led to IF were multiple. In line with other reports, the most frequent condition was intestinal ischemia [1,18]. Some of them were rare and posed significant diagnostic challenges (e.g., CIPO and hypogammaglobulinemic sprue from rituximab therapy) [19].

One of the most notable outcomes was the nutritional improvement, as evidenced by a statistically significant increase in BMI during the follow-up period. At the beginning of HPS, 65.2% of patients were underweight, dropping to 17.4% by the end of HPS/follow-up. Moreover, none of the patients reached obesity, indicating that the caloric intake provided by HPS can effectively meet each patient’s energy requirements without overfeeding. The results emphasize the efficacy of HPS in addressing malnutrition and stabilizing the nutritional status of IF patients.

Weaning off HPS in parallel with a progressive increase in enteral autonomy is part of the treatment goal [4]. The overall percentage of weaning from HPS is around 50% in 2 years, but the recovery of intestinal autonomy after this period is still possible [20]. This recovery is supported by mechanisms like intestinal adaptation, colonic hyperfermentation with short-chain fatty acid production and hyperphagia [21,22,23,24,25]. In recent years, therapy with GLP-2 analogs has been shown to reduce HPS requirements and potentially lead to complete weaning off in some patients because of its mucosal trophic effects, enhancing intestinal adaptation [26,27,28,29]. Teduglutide was the first approved GLP-2 analog. Typically, it is used in stable patients who cannot be weaned from HPS despite all other strategies. The use of GLP-2 analogs has been growing and has the potential to revolutionize the management of IF patients [29]. Since GLP-2 analogs act as growth factors, they are contraindicated for patients with active or recent malignancies (<5 years) [30,31]. This contraindication limits the broader use of this therapy, especially given that our hospital is a reference center for colorectal cancer, and a significant portion of our patients develop short bowel syndrome as a result of surgical complications from this pathology. Nevertheless, in our center, a considerable portion of patients were completely weaned off HPS. Two patients were treated with Teduglutide. One of the patients was already in the weaning process but could only reach complete weaning off HPN after 6 months of Teduglutide administration. At the moment of data collection, a patient with a past medical history of gynecological cancer but with no evidence of recurrence after 5 years started this therapy.

Although HPS can result in an annual mortality rate excess of 1% [20], long-term survival has improved significantly over the decades. We achieved a considerable mean survival of 43.4 months. In our study, 43.5% of the patients died during the follow-up period, but only one death (4.35%) was directly attributable to HPS-related complications. These data reflect the efficacy and safety of HPS.

Despite positive outcomes, HPS was not free from complications. CRBSI was the most frequent complication, with an incidence rate of 2.5 per 1000 catheter days. The reported incidence in the literature varies widely, from 0.2 to 11.5 cases per 1000 catheter days [32,33]. This wide range reflects differences in patients (such as underlying conditions) and center-dependent factors (such as specific protocols and experience). However, the criteria used to diagnose CRBSI play a major role in this variety. According to the European Society for Clinical Nutrition and Metabolism (ESPEN) criteria, CRBSI is diagnosed by positive paired central and peripheral blood cultures isolating the same micro-organism (same species and antibiograms) in a patient with clinical symptoms/signs of bloodstream infection and no other apparent source of infection [1]. In the daily practice, a more clinical approach is frequent, allowing diagnosis based on symptoms/signs of CRBSI and no other apparent source of infection even with no positive paired blood cultures. A clinically based diagnosis has been shown to lead to an overdiagnosis of almost 50% compared to a strict microbiological strategy [34]. In our study, we classified cases as CRBSI even without positive paired blood cultures (or even without an isolated micro-organism in 6.1% of patients), although most cases fulfilled the ESPEN criteria. Gram-positive bacteria and particularly *Staphylococcus aureus* were the most isolated pathogens, consistent with the reported literature [33]. Fungal infections, notably the *Candida* species, were also a significant concern, occurring in 16.3% of cases. Occasionally, these infections led to more severe complications, such as acute endocarditis, underscoring the importance of infection prevention measures. Metabolic complications requiring hospitalization were the second most frequent group of complications, with an incidence of 1.2/1000 catheter days.

Comparing HPS-related complications before and after the legal paradigm change, which allowed HPS to be self-administered by the patients at home, its incidence was similar (overall complications even displayed a small decrease). This was only possible due to our multidisciplinary team’s efforts to effectively train and educate our patients, families and/or caregivers to safely implement HPS provision, and this may be considered a surrogate marker of the quality of our training.

An effective management of patients with intestinal insufficiency is crucial in preventing the progression to IF. In fact, our multidisciplinary team follows a large number of patients with intestinal insufficiency, providing tailored care that is essential to prevent the need for IVS. The measures include personalized oral and/or enteric nutrition plans to maximize the remaining intestinal function, along with pharmacological treatments and surgical interventions to manage the underlying conditions and complications while providing ongoing care and education to patients and their families. Ultimately, this proactive approach also reduces the number of IF patients followed in our center.

The prevalence of IF patients requiring HPS in Portugal is reported to be very low compared to other European countries [16,35,36]. Rather than representing a truly low prevalence, this is believed to reflect the major difficulties that Portuguese patients had been facing in accessing this life-saving therapy due to no legal support for it, as well as the complete absence of national registries in this field. Nevertheless, our team gained earlier experience with home parenteral support due to our hospital’s pioneering role in domiciliary hospitalization. Currently, a turning point is being marked by the Portuguese national health directive 017/2020 and the ongoing organization of a Portuguese chronic intestinal failure registry, bringing HPS in Portugal into a new era [16,17]. We foresee a significant increase in our experience in the near future.

## 5. Conclusions

The underlying conditions leading to IF were diverse, with SBS being the most frequent pathophysiological cause. HPS significantly improved the nutritional status, as reflected by a statistically significant BMI increase, while none of the patients reached obesity. These nutritional outcomes emphasize the critical role of HPS in addressing malnutrition without overfeeding. The most common complications were CRBSI, mainly via Gram-positive bacteria, with *Staphylococcus aureus* being the most frequent pathogen. Despite common complications leading to multiple hospitalizations, HPS demonstrated a high safety profile and allowed considerable survival, with only one death directly attributable to a HPS-related complication. These results highlight the safety and effectiveness of HPS in the management of IF, despite these being the first patients of a previous unexperienced team.

Emerging therapies like GLP-2 analogs show promising results in reducing HPS dependence and are revolutionizing IF treatment. HPS in Portugal lags significantly behind other European countries. Currently, it is reaching a turning point, and we expect an increasing development in the near future. We emphasize the need for standardized national data registries and the establishment of specialized IF centers, particularly in countries like Portugal, where IF remains underreported, and its management remains poorly organized. Our present report illustrates how the lag of the Portuguese panorama has allowed us to benefit from the great European experience for a more solid initial development.

## Figures and Tables

**Table 1:** Intestinal failure: functional, clinical and pathophysiological classifications.

Functional Classification					
Type I	Acute and short-term. Resolves spontaneously along with the resolution of its underlying cause. Most often seen post-operatively, requiring IVS for a few days.				
Type II	Prolonged subacute. Most often seen in metabolically unstable patients with a high-output stoma or enteric fistulas, requiring IVS for weeks or months. Often progresses to type III.				
Type III	Chronic condition in metabolically stable patients, requiring IVS for months or years. Can be reversible or irreversible.				
Clinical Classification					
		volume of IVS (mL/day)			
		≤1000 (1)	1001–2000 (2)	2001–3000 (3)	>3000 (4)
Type of IVS					
Fluids and electrolytes (FE)		FE1	FE2	FE3	FE4
Parenteral nutrition (PN)		PN1	PN2	PN3	PN4
Pathophysiological Classification					
Short bowel Intestinal fistula Intestinal dysmotility Mechanical obstruction Extensive small bowel mucosal disease					

**Table 2:** Patient characteristics and distribution according to functional, severity and pathophysiological classifications.

Sex	N (%)
Female	12 (52.2)
Male	11 (47.8)
Age	
<65 years	15 (65.2)
≥65 years	8 (34.8)
Type of HPS	
HPN	18 (78.3)
HPH	5 (21.7)
Functional classification	N (%)
Type III	23 (100)
Severity classification	
HPH patients	
FE1	3 (13.0)
FE2	2 (8.7)
HPN patients	
PN1	1 (4.4)
PN2	11 (47.8)
PN3	6 (26.1)
Pathophysiological classification	
Short bowel	16 (69.6)
Type 1	14 (60.9)
Type 3	2 (8.7)
Intestinal fistula	4 (17.4)
Intestinal dysmotility	2 (8.7)
Extensive small bowel mucosal disease	1 (4.3)

**Table 3:** Microbiological agents of infectious complications.

Microbiological Agents	N (%)
Gram-positive bacteria	29 (59.2)
*Staphylococcus aureus*	10 (34.5)
*Staphylococcus epidermidis*	9 (31.0)
*Enterococcus faecalis*	4 (13.8)
*Staphylococcus haemolyticus*	3 (10.3)
*Staphylococcus lugdunensis*	2 (6.9)
*Staphylococcus capitis*	1 (3.5)
Gram-negative bacteria	9 (18.4)
*Enterobacter cloacae*	3 (33.3)
*Klebsiella oxytoca*	2 (22.2)
*Klebsiella pneumoniae*	2 (22.2)
*Pseudomonas aeruginosa*	1 (11.1)
*Kluvyera ascorbata*	1 (11.1)
Fungi	8 (16.3)
*Candida parapsilosis*	6 (75)
*Candida albicans*	1 (12.5)
*Candida guilliermondi*	1 (12.5)
No microbiological agent identified	3 (6.1)

**Table 4:** HPS-related complications.

	Total (Per 1000 Catheter Days)	Before Paradigm Shift (Per 1000 Catheter Days)	After Paradigm Shift (Per 1000 Catheter Days)
Overall	4.3	4.5	4.1
Infectious	2.5	2.5	2.5
Metabolic	1.2	1.1	1.2
Dysfunction/ Exteriorization	0.4	0.7	0.2
Thrombotic	0.3	0.2	0.3

## Data Availability

The data presented in this study are available upon request due to ethical reasons.
